# Is acupuncture “stimulation” a misnomer? A case for using the term “blockade”

**DOI:** 10.1186/1472-6882-13-68

**Published:** 2013-03-25

**Authors:** Morry Silberstein

**Affiliations:** 1 Faculty of Science & Engineering, Curtin University, GPO Box U1987, Perth, WA 6845, Australia

**Keywords:** Acupuncture, Neural blockade, Capsaicin, Low level laser therapy, Alpha-2 adrenoceptor

## Abstract

**Background:**

The term used most frequently in the literature to describe acupuncture’s effects is “stimulation” which may be used to describe either (or both) the direct stimulus applied to a needle as well as putative stimulation of the nervous system, despite little published evidence describing what is actually being stimulated. In contrast, recent published work has suggested that acupuncture may, in fact be inhibitory at a peripheral level, acting by blocking neural transmission.

**Discussion:**

The suggestion that acupuncture exerts its effects through peripheral neural blockade is supported by recent evidence explaining related techniques including low level laser and capsaicin at acupoints. It also explains acupuncture’s effect on painful and non-painful conditions and both Eastern and Western concepts of acupuncture. There is a need for additional work to elucidate acupuncture’s mechanism of action, and the suggestion that it acts through neural blockade should prompt further research in this direction.

**Summary:**

If the term “blockade” were applied to acupuncture, this would, likely, be expected to promote this minimally invasive technique, and, potentially, bring it into mainstream clinical practice for pain management as well as other therapeutic applications.

## Background

While the exact mechanism by which acupuncture exerts its clinical effect remains unclear, the description used for elicitation of this effect is widely termed “stimulation”. Indeed, a National Library of Medicine (PubMed) search for “acupuncture” conducted by the author on December 31, 2012 identified 18,549 articles, of which 3,334 (18%) incorporated the term “stimulation”. In contrast, only 372 (2%) incorporated the term “inhibition” and a mere 57 (0.3%) included the term “blockade”. This terminology appears to be based upon acupuncture’s historical association with Traditional Chinese Medicine, which deems that this technique stimulates acupoints along meridians to restore their function through the regulation of yin, yang and qi [1]. Acupuncture has certainly been shown to stimulate the production of a wide variety of humoral mediators, including opiates [2], monoamines [3], corticoids [4] and purines [5] by the nervous system. While it is unclear as to what proportion of these 3,334 articles used the term “stimulation” to mean direct stimulus (manual or electrical) applied to a needle, as opposed to putative stimulation of the nervous system, the use of this term is widespread, and its ongoing use merits further consideration and clarification.

Indeed, there is recent evidence that acupuncture, and, especially, related therapeutic modalities such as low level laser therapy and capsaicin plaster – both at acupuncture points – exert their effects through neural blockade. Indeed, both laser and capsaicin appear to act at the skin level through an inhibitory mechanism. Low level laser – when applied to acupuncture points – appears to exert equivalent effects to needle acupuncture [6], and has been shown to be a highly effective therapeutic technique for pain [7]. Yet, rather than being stimulatory, Chow et al. have found that laser actually inhibits peripheral nerves [8]. Similarly, the application of capsaicin to acupuncture points in bother upper [9] and lower [10] limbs results in identical effects to needle acupuncture, yet capsaicin induces neural conduction blockade rather than stimulation [11]. It is, of course, quite possible, that acupuncture employing needle insertion induces its clinical effect through an entirely different mechanism to that of laser acupuncture and capsaicin application to acupuncture points. Nonetheless, is it not worth considering whether the therapeutic mechanism of these three techniques might be identical or comparable?

Zunhammer et al. have recently demonstrated that verum – but not sham – needle acupuncture inhibits motor excitability [12]. How would “stimulation” inhibit muscle tone? If acupuncture were stimulatory at the periphery, would a second “stimulation” not be expected to increase brain activity? Yet,Yeo et al. found that consecutive needle acupuncture treatments at the same point resulted in reduced functional MRI brain activation following the second treatment 5 minutes after the first [13]. Neuropeptide Y is released by the hypothalamus in response to, and is a marker of, physical or metabolic stress [14], yet Eshkevari et al. have recently demonstrated that needle acupuncture blocks this response [15], and there is also recent evidence suggesting that acupuncture blocks the expression of transient receptor potential neuronal ion channels [16].

Could a case be made for replacing the term “acupuncture stimulation with the term “acupuncture blockade”?

## Discussion

The mechanism by which acupuncture exerts its clinical effect remains unclear, but the term “stimulation” remains in wide clinical use [1]. If acupuncture is stimulatory, what is actually being stimulated? The term “stimulation” in acupuncture may be used to describe either (or both) the direct stimulus applied to the needle (for needle acupuncture), as well as putative stimulation of the nervous system that is assumed to derive from the applied mechanical or electrical needle stimulation. While there is certainly evidence indicating that some forms of acupuncture result in stimulation of endogenous mediators, especially opiate production following electroacupuncture [2], the eminent Chinese acupuncture researcher, Professor JS Han, wrote in 2004 (in relation to the stimulation of endorphin release), “The findings obtained from experimental animals need to be confirmed in humans in clinical practice [2].” Opioid release continues to be shown to be involved in the mechanism of electroacupuncture analgesia in experimental animals, most recently by Meng et al. in 2011 [17], but has yet to be confirmed as being involved in humans, while only a solitary published study exists in support of laser acupuncture causing endogenous opioid release, that study utilizing power outputs substantially far greater than those used in clinical practice [18].

If acupuncture works by blocking – rather than stimulating – nerves, in addition or an alternative to endogenous mediator release, where does this occur, and is this blockade a peripheral or central effect? Using functional brain imaging, Hsieh et al. demonstrated an increase in periaqueductal gray cerebral activation following painful stimulation [19], while Zyloney et al. later showed that acupuncture at verum – but not sham – points blocked periaqueductal gray activation [20]. Hence, acupuncture may well block the brain activation induced by a painful event, but is this central blockade “direct” or “secondary”? Any primary afferent nerve stimulation can potentially elicit a complex pattern of secondary CNS responses, some excitatory and some inhibitory [21], but is there a unifying peripheral mechanism by which application of a needle, laser or capsaicin plaster might cause an inhibitory response?

Low level laser appears to block peripheral nerves by suppressing neural action potentials through induction of mitochondrial dysfunction and the disruption of microtubule arrays and fast axonal flow [8], while capsaicin results in vanilloid receptor desensitization, also blocking peripheral nerves [11]. How might needle or electroacupuncture block peripheral nerves? One possible explnantion previously suggested by the author is that acupoints are the branch sites of unmyelinated cutaneous afferents, and that acupuncture disrupts this point, blocking both centripetal sensory transmission to the spinal cord and tangential communication along sites of peripheral afferent cross-talk [22]. Preliminary evidence in support of this theory has since been found in histologic analysis of human acupuncture points [23]. Langevin and colleagues have shown that the fascia surrounding a needle inserted at an acupuncture point “grasps” the needle, and suggested that there is winding and stretching of the fascia around the needle as it is rotated [24]. There are recently-discovered stretch-activated transient receptor potential (TRP) ion channels on nociceptive axons [25], and their stretching during needle acupuncture may well case desensitization and blockade, in a manner similar to capsaicin at vanilloid TRP channels. Similarly, electrical stimulation of C-fibers, as might occur in electroacupuncture, results in prolonged conduction blockade [26].

If acupuncture were to act primarily by stimulating release of endogenous opiates or other mediators, then point selection should be irrelevant. Langevin and colleagues have demonstrated that over 80% of acupuncture points are located along connective tissue cleavage planes [27], and, subsequently, fMRI studies have shown that acupuncture at known acupoints results in a very different response to acupuncture at non-acupoint sites [28-30]. If point selection is relevant to the mechanism of acupuncture’s effect, this would be at odds with the suggestion that it works by stimulating endogenous opiate or other mediator release.

This might indicate that precise acupuncture point localization prior to needle insertion could be critical for eliciting a specific response, yet there is evidence of considerable imprecision in needle placement [31], and Habib [32] has recently suggested that this imprecision may account for the variability in the results of acupuncture trials. For acupuncture to work by directly disrupting – and thus blocking – nerves emerging at connective tissue cleavage planes, there would need to be considerable accuracy in point localization.

The proposal that acupuncture blocks nerves at a peripheral level would uniquely explain three major issues which have previously not been well understood:

### The multitude of modalities used in clinical acupuncture

As noted above, acupuncture treatment is not strictly limited to needle insertion, with many practitioners employing other techniques, including moxibustion [1], low level laser [6], capsaicin plaster [9], and even simple manual acupoint massage (acupressure) [33].

Yet, all of these techniques are centered upon treating specific acupoints, and in common with needle acupuncture, can be understood if considered to block neural conduction peripherally [8,11,22].

### Acupuncture’s range of clinical effects

In addition to its use in treating pain, acupuncture has been shown to be a useful therapeutic technique for a range of clinical conditions including nausea [34], depression [35], insomnia [36], and immunosuppression [37]. A *stimulatory* mechanism explaining acupuncture’s effects in these and other non-pain related conditions has yet to be described, but there is certainly recent evidence that, for insomnia, at least, acupuncture is inhibitory [38]. Is there a unifying *inhibitory* mechanism through peripheral neural blockade which might explain acupuncture’s wide-ranging effects on conditions other than pain? Acupuncture’s effect on nausea is believed to involve gastric motility enhancement [39]. The stimulation of intestinal alpha-2 adrenoceptors inhibits gut motility [40], while the administration of alpha-2 adrenoceptor antagonist yohimbine results in enhanced motility [41]. If acupuncture blocked the C – fiber reflex known to activate visceral sympathetic efferents to the gut [42], this would result in enhanced motility through alpha-2 adrenoceptor blockade. Similarly, Yanpallewar et al. have demonstrated that alpha-2 adrenoceptor blockade has a potent anti-depressant effect [43], while alpha-2 adrenoceptor blockade has been shown to have a potent anxiolytic effect [44], which could readily be extrapolated to insomnia. In relation to immune modulation, alpha-2 adrenoceptor stimulation has been show to induce immunosuppression [45], while blockade of these receptors results in immune enhancement [46]. Hence, acupuncture’s effect on both painful and non-painful conditions can be explained by invoking neural blockade, either directly or via secondary blockade of sympathetic efferents that activate alpha-2 adrenoceptors.

### Eastern and Western concepts of acupuncture

Eastern concepts of acupuncture invoke unblocking sites of impaired energy flow along meridians caused by conditions such as stagnationof energy [1]. While this might explain acupuncture’s local and distant effects in Traditional Chinese Medicine (TCM), there appears to be no obvious scientific correlate to meridians [47]. In contrast, Western concepts attribute acupuncture’s local effects to Aβ fibre skin stimulation inhibiting painful stimuli from the periphery [47], thus reducing pain perception, based upon local dermatome selection [48]. Yet, this does not adequately explain distant effects, with the suggestion that acupuncture stimulates descending inhibition through activation of diffuse noxious inhbitory controls having recently shown to be unlikely [49], and other invoked mechanisms, such as release of endogenous opiates, not requiring acupoint specificity [47]. There is, however, evidence of convergence of spinal afferent processing between apparently unrelated viscera, which have specific parallels in TCM approaches. For example, Eastern concepts of acupuncture which pair lung and colon meridians [1] have correlates with recently demonstrated spinal cross-talk between lung and colon [50]. If meridians, as recently suggested [51], are subepidermal C – fiber afferents, then acupuncture blockade could be expected to have both local and distant effects, as well as influence apparently unrelated viscera such as lung and colon.

Hence, a case could be made for replacing the term “acupuncture stimulation” with “acupuncture blockade”. What are the implications for acupuncture practitioners of using the term “acupuncture blockade”? The alignment of acupuncture with other anesthetic techniques for neural blockade would, likely, be expected to promote this minimally invasive technique, and, potentially, bring it into mainstream clinical practice for pain management as well as other therapeutic applications. In addition, there would be substantial impetus for further research to finally elucidate acupuncture’s mechanism of action.

## Summary

While acupuncture has previously been deemed to be stimulatory, there is little published evidence to support using this term for the peripheral effect of the needle, laser or capsaicin used to induce the clinical effect. In contrast, there is recent evidence to suggest that acupuncture acts through an opposing peripheral mechanism – neural blockade – with this mechanism explaining the range of acupuncture techniques in use, its wide clinical application and both Eastern and Western concepts of acupuncture. Hence, it is suggested that consideration be given to acupuncture “stimulation” being re-named acupuncture “blockade”.

## Competing interests

The author declares that he has no competing interests, either financial or non-financial in relation to this manuscript.

## Authors’ contribution

The author contributed to all aspects of the manuscript and was unassisted in this contribution.

## Pre-publication history

The pre-publication history for this paper can be accessed here:

http://www.biomedcentral.com/1472-6882/13/68/prepub
